# Depressive symptoms in parents are associated with reduced empathy toward their young children

**DOI:** 10.1371/journal.pone.0230636

**Published:** 2020-03-23

**Authors:** Virginia C. Salo, Sara J. Schunck, Kathryn L. Humphreys

**Affiliations:** Department of Psychology and Human Development, Vanderbilt University, Nashville, Tennessee, United States of America; International Telematic University Uninettuno, ITALY

## Abstract

**Background:**

While depression is typically associated with reduced levels of empathy, this association differs depending on how empathy is measured. Given the importance of empathy in the parent–child relationship, we sought to examine whether the associations between depression and dispositional empathy would also extend to empathy towards one’s own child.

**Methods:**

Within a non-clinical sample of 150 parents of young children, we examined the associations between self-reported depressive symptoms, dispositional empathic tendencies, and empathy specifically toward their own children, and how these associations might differ based on parent gender.

**Results:**

Depressive symptoms were related to lower levels of cognitive and affective empathy, and higher levels of empathic distress. Over and above the association with dispositional empathy, depressive symptoms were associated with reduced levels of parents’ affective empathy toward their own child. The associations between depressive symptoms and both dispositional and own-child specific empathy varied by parent gender. For fathers, depressive symptoms predicted own-child specific affective empathy, over and above dispositional affective empathy, while for mothers, depressive symptoms predicted own-child specific empathic distress, over and above dispositional empathic distress.

**Conclusions:**

The current findings provide further indication that caregivers with elevated depression may engage in patterns of thoughts and behaviors that have implications for their interactions with their children. Parents’ experienced empathy toward their own child may be one mechanism by which depression impacts the early caregiving environment, and thus may be an important target for intervention in improving the early caregiving experiences for children at elevated risk due to parental depression. Differences in cognitive and affective empathy found among those with depression may be even more pronounced in the thoughts and feelings towards one’s own child, making this an important clinical target.

## Introduction

The first few years of a child’s life are marked by sensitivity to the environment, and as such the interactions a child has with their caregivers during this time provides a rich source of early learning that is theorized to have lasting effects on development across domains [1–3]. Depression in parents is a significant risk factor for poorer outcomes for children, in part due to differences in the parent–child relationship [4]. In the current study, we examine how parental depressive symptoms might impact their feelings of empathy toward their children as one potential explanation for how parental depression can impact the parent–child relationship.

Across the entire world population, more than 300 million people are estimated to experience a major depressive episode in their lifetime [5]. Among adults in the United States alone the lifetime prevalence of major depressive disorder (MDD), according to DSM-5 [6] criteria, is approximately 21% [7]. One study that considered subclinical depressive symptoms, in line with DSM-3 criteria, in adults in the Netherlands reported a prevalence rate of about 11% in addition to those with clinically significant depressive symptoms [8], and a national survey in the UK, using the revised Clinical Interview Schedule (CIS-R) [9], estimated approximately 15% of adults may experience significant depressive symptoms at a given time [10]. Using a short form of the Center for Epidemiologic Studies Depression Scale (CES-D) [11], items on which align with DSM criteria, to assess depressive symptoms in a representative sample of the United States, Paulson and colleagues [12] found high rates of postpartum depression in both mothers (14%) and fathers (10%) when their children were age 9 months. Importantly, depression in parents is a significant risk factor for poorer outcomes for children [13,14]. For example, maternal depression is negatively associated with a child’s language development [15,16]. Children of depressed mothers exhibit a stronger physiological response to stress (as measured by increased cortisol levels) [17–20]. Parental depression is also positively associated with the development of internalizing and externalizing problems [21,22] and increased risk for meeting diagnostic criteria for psychiatric disorders [23]. Further, persistent depressive symptoms in parents is associated with increased risk for adverse child outcomes [24].

Increased risk for negative outcomes among the offspring of parents with depression may be due to many potential factors [13,14]. In addition to potential shared genetic risks and differences in the prenatal environment, there is robust evidence that the offspring of depressed and non-depressed parents differ in the caregiver experiences they receive [4]. Parental depression is associated with greater negative parenting behaviors (e.g., neglect, aggression) and lower levels of positive parenting behaviors (e.g., sensitivity, engagement, warmth) [25]. These patterns are found whether depression is measured in terms of symptoms or diagnosis [26], in analyses of both small and large datasets [27], and in studies of maternal or paternal depression [28,29]. Further, depressive symptoms are negatively associated with physical care practices in mothers (e.g., feeding and sleep practices), and negatively associated with engagement in cognitively enriching activities (e.g., reading) in both mothers and fathers. Fathers with postpartum depression exhibit lower levels of responsive and sensitive parenting [30]. Dix and colleagues found evidence that offspring demonstrate a learned pattern of emotional withdrawal from mothers who are often unresponsive [31]. Depression may also impact parents’ capacity to engage in appropriate dialogue with their children about their emotions [32]. Further, the association between maternal depression and child problem behaviors can be explained, in part, by differences in maternal parenting behaviors [33].

One explanation for why parents with depression might exhibit differences in their parenting behavior comes from research on the cognitive patterns associated with depression. Depression is associated with aberrant and often negative cognitive patterns [34]. In particular, individuals with depression tend to exhibit a more egocentric world-view [35] and increased self-focus [36]. As such, several studies have examined whether there exists an association between depression and empathy, the ability to interpret and relate to the feelings and emotions of others, which is seen as a critical foundation for adaptive social functioning [37].

Empathic abilities are often broken down into three unique components, based on the dimensions outlined by Davis [38,39]. *Cognitive empathy*, or perspective taking, refers to one’s tendency to recognize emotion states in others and reason about their cause or consequences; *affective empathy*, or empathic concern, refers to one’s tendency to sympathize with others’ feelings; and *empathic distress* refers to the tendency to experience your own distress or anxiety in response to others’ emotions or circumstances. The first two components are importantly distinct from the third in that they reflect an ‘other-oriented’ response, requiring psychological resources to be directed toward others and away from the self [40]. As Decety and Jackson [41] outline, these three components likely reflect and are supported by unique cognitive processes, with cognitive empathy representing top-down processing, affective empathy drawing on more bottom-up processing, and empathic distress reflecting a continuum of self-regulatory processes.

There is evidence for some dissociation of deficits across these different dimensions in individuals with mood and other psychiatric disorders [42,43]. One study found current MDD diagnosis was related to reduced levels of both cognitive and affective empathy, as compared to individuals without MDD, however no differences were found in terms of empathic distress [44]. In a Dutch sample, Bennik, Jeronimus, and aan het Rot [45] found depressive symptoms were associated with reduced cognitive empathy, and a positive but weak association between depressive symptoms and affective empathy. Alternatively, another study found depressive symptoms to be positively associated with empathic distress, but unrelated to either cognitive or affective empathy [46]. In line with this last finding, depression has also been linked with a failure to maintain a self-other distinction [47,48], which might explain the increased empathic distress. According to this framework, cognitive or affective empathy deficits in individuals with depression may reflect a self-preservation response to impairment from the heightened arousal they experience in reaction to others’ negative emotions. Indeed, decreased cognitive empathy is associated with depression specifically in situations that require inhibition of one’s own emotional state [49], and feelings of guilt and shame have been shown to mediate the associations between depressive symptoms and affective empathy [50]. Taken as a whole, these findings suggest that depression is associated with differences in empathic responses, however the specificity of these associations is remains unclear.

### Current study

In the current study, we sought to replicate and extend research on the association between depression and empathy. Specifically, we aimed to further examine the association between depressive symptoms and the three components of dispositional empathy, as assessed through the Interpersonal Reactivity Index (IRI; 39). Further, in a limited body of literature, parents’ dispositional empathic tendency is found to be related to both parenting behaviors and child outcomes [51,52]. Mothers who report lower levels of dispositional empathy are at a higher risk for child neglect [53,54]. Importantly, parental empathy, that is, empathy specific to the parent–child relationship, is found to be associated with children’s attachment security [55]. Given the importance of caregiver empathy towards their child and the documented links between parental depression, parenting behavior, and child outcomes, we sought to determine whether the expected deficits in dispositional empathy associated with depressive symptoms would also extend to empathy towards one’s own child. In addition, while maternal, relative to paternal, depression is reported to have a greater impact on children [56–58], depression in fathers is also associated with increased risk for offspring behavioral problems and psychopathology [59]. Additionally, the association between paternal depression and negative child outcomes exists above and beyond the more well-known negative effects of maternal depression [60]. Thus, we also sought to determine whether anticipated associations between depressive symptoms and empathy, either dispositional or own-child specific, were consistent across mothers and fathers.

Our aims were three-fold. First, we sought to examine the associations between depressive symptoms and dispositional empathy. Based on the evidence reviewed above suggesting links between depression and the three dimensions of empathy [44,45], we anticipated that higher levels of depressive symptoms would be uniquely associated with all three dimensions of empathy. Specifically, we expected that depressive symptoms would be negatively associated with cognitive and affective empathy and positively associated with empathic distress. Second, we sought to explore the associations between depressive symptoms and empathy specifically toward one’s own child. As this question has yet to be addressed in the literature, we based our hypotheses on the evidence for differences in parenting behavior associated with parents’ experience with depression [e.g., 25], and in particular those studies suggesting a level of emotional withdrawal from their children in parents with depression [31,32]. We expected that depressive symptoms would be related to decreased empathy for one’s own child, and we further explored whether this association would persist over and above the association with dispositional empathy. Third, we sought to investigate how associations between depressive symptoms and empathy might be consistent or might differ across mothers and fathers, about which we made no specific hypotheses. To address these aims, we assessed depressive symptoms and empathy in a sample of mothers and fathers with young children. Importantly, we used the IRI [39] to assess dispositional empathy as well as a modified IRI to assess empathy specific to their own child [61]. It should also be noted that the current study utilizes cross-sectional self-report data, therefore our ability to generate causal explanations about these anticipated relations is limited and we thus focus our analyses and conclusions on the concurrent relations among the constructs in question.

## Methods

### Participants

Participation in the study was conducted through Amazon’s Mechanical Turk (MTurk) platform. This study was approved by the Stanford Institutional Review Board (eProtocol #40302). Written informed consent was obtained from all individual participants included in the study. Eligibility criteria for the current study were as follows: participants were required to be a U.S. or Canada resident, have advanced English language proficiency, and have at least one child between the ages of 12 and 36 months. Participants were compensated the equivalent of $8/hour to participate. Consent was provided by signing an online form following a thorough description of the eligibility criteria, study goals, and study procedures. The questions and analyses presented here were developed post-hoc to the study design, as such the sample included participants form two different waves of data collection each of which included a different set of questionnaires, but which overlapped in those measures of interest for the current study. Only participants who had complete data for all measures used in the current study were included in the analyses. Due to concerns regarding “bots” and “farmers” on Mechanical Turk [62], we removed respondents who incorrectly responded to a check question as well as those with exact matches and repeats for IP address or geolocation (latitude and longitude), a screening technique which has been shown to effectively eliminate invalid responses [62,63]. Two hundred and ten valid responses remained after this screening. Sixty participants were then excluded for not meeting eligibility criteria (n = 6), or having incomplete data (n = 54). The final sample consisted of 150 parents (44% female) who ranged in age from 23 to 46 years (M = 33.49, SD = 5.48). Demographic information is presented in Table 1.

**Table 1:** Participant demographic information.

Variable	n	%
Gender		
Male	84	55
Female	66	44
Number of Children		
One	91	61
Two	38	25
Three+	21	14
Race		
White/Caucasian	116	77
Black/African-American	15	10
Asian	14	9
American Indian or Alaska Native	3	2
Two or more	1	1
No response	1	1
Ethnicity		
Hispanic	10	7
Not Hispanic	140	93
Marital Status		
Married or partnered	125	83
Single	19	13
Divorced	3	2
Separated	2	1
Widowed	1	1
Education		
Graduate degree	29	19
Bachelor’s degree	64	43
Associate’s degree	19	12
Some college	16	11
High school diploma	16	11
Some high school	1	1
Technical/vocational training	5	3

### Measures

#### Dispositional empathy

Dispositional empathy was evaluated by the IRI [39]. The IRI consists of 28 items total, with seven items each corresponding to four subscales for empathic concern, perspective taking, personal distress, and fantasy. The seven fantasy items were excluded for the purpose of this study because there was no equivalent subscale on the own-child specific empathy measure, described below, and it did not align with one of the three primary dimensions of empathy, described above. The remaining 21 items were answered on a five-point Likert-type scale ranging from 0 (does not describe me well) to 4 (describes me very well). The empathic concern subscale assesses feelings of concern and sympathy towards others (e.g., “I am often quite touched by things that I see happen”) and reflects the construct of affective empathy. The perspective taking subscale measures the tendency to take another’s psychological point of view (e.g., “When I’m upset at someone, I usually try to ‘put myself in his shoes’ for a while”) and reflects the construct of cognitive empathy. The personal distress subscale determines feelings of self-oriented anxiety when others are in distress (e.g., “I sometimes feel helpless when I am in the middle of a very emotional situation”) and reflects the construct of empathic distress. Higher scores on each of the three subscales indicate greater levels of self-reported empathy within the respective domain. The factor structure underlying the four subscales has been shown reliable across multiple samples [64], and the scale also exhibits strong test-retest reliability (all subscale *r*s > .61) [38,39]. Scores on the IRI also show strong convergence with other measures of empathy [38]. Further, the strong psychometric properties of the IRI have repeatedly been confirmed across many samples [65–67]. Internal consistency for the IRI was high in the sample for the whole measure (Cronbach’s α = .83), and within each subscale (empathic concern α = .85; perspective taking α = .83; personal distress α = .85). Descriptive statistics are presented in Table 2.

**Table 2:** Correlations between and descriptive statistics for subscales of dispositional and own child empathy.

	**Dispositional**			**Own-Child**		
	Empathic Concern	Perspective Taking	Personal Distress	Empathic Concern	Perspective Taking	Personal Distress
**Dispositional**						
Empathic Concern	1	.70***	-.12	.65***	.53***	-.08
Perspective Taking		1	-.05	.46***	.59***	.08
Personal Distress			1	-.27**	-.13	.73***
**Own-Child**						
Empathic Concern				1	.59***	-.21**
Perspective Taking					1	-.07
Personal Distress						1
**Mean (SD)**	20.25 (5.57)	18.44 (5.41)	9.69 (5.99)	23.09 (5.14)	19.33 (5.91)	11.09 (6.13)
**Range**	6–28	8–28	0–27	8–28	5–28	0–26

*p < .05;

**p < .01;

***p < .001; n = 150.

#### Own-child empathy

Parents’ empathy for their own child was measured using the Interpersonal Reactivity Index—Parent Empathy (IRI-PE; [61]), an adapted version of the IRI made specifically to target parents’ empathy for their children. The 21 items from the empathic concern, perspective taking, and personal distress subscales were revised to ask about the participant’s own child. For example, the IRI item “Sometimes I don’t feel very sorry for other people when they are having problems” became “Sometimes I don’t feel very sorry for my child when (s)he is having problems” for the IRI-PE. Items were answered on a five-point Likert-type scale ranging from 0 (does not describe me well) to 4 (describes me very well). Participants were asked to think about their child age 12–36 months. If any participant had more than one child is this age range, they were instructed to answer these questions in relation to their youngest child within the range. As with the IRI, higher scores on each of the three subscales indicate greater levels of self-reported empathy within the respective domain. To our knowledge, cross-sample psychometric properties have not yet been reported for the IRI-PE, however internal consistency was similarly high for the IRI-PE (α = .79; empathic concern α = .81; perspective taking α = .86; personal distress α = .81). Further, previous work using the IRI-PE showed scores on the empathic concern and perspective taking subscales are positively associated with other measures of empathic responding [61]. Descriptive statistics are presented in Table 2.

#### Depressive symptoms

The Center for Epidemiologic Studies Depression Scale (CES-D) was used to measure participants’ depressive symptoms. The CES-D includes items relating to depressive symptoms on multiple dimensions (depressed affect, positive affect, somatic symptoms/ retarded activity, and interpersonal). Each question asks participants about the frequency of feelings and behaviors within the time frame of “the past week”. Responses are on a four-point Likert-type scale ranging from 0 (rarely or none of the time [less than 1 day]) to 3 (all of the time [5–7 days]). Responses are summed across all items; thus, higher scores are indicative of a greater level of depressive symptoms. Two forms of the CES-D were used in the current study. The 20-item CES-D (CESD-20; [11]), on which scores can range from 0–60, was administered to 115 participants, and had a high internal consistency in this sample (α = .93). When the second subset of data was collected, in order to reduce participant burden, the shorter 10-item CES-D (CESD-10; [68]) was completed by the remaining 35 participants. This short form consists of 10 items taken directly from the original 20-item form with possible scores ranging from 0–30, and also had a high internal consistency in this sample (α = .88). Both versions of the CES-D have evidenced strong psychometric properties in assessing symptoms of depression [11,68]. The CESD-20 is highly correlated with scores on other self-report measures of depressive symptoms [69] and shows strong predictive reliability for clinical diagnosis [70]. The CESD-10 has a retest reliability of *r* = .59 and is accurate in predicting scores on the CESD-20 [68].

To create a common depressive symptoms measure that utilized the full variability of the 115 participants who had completed the longer form, the following steps were taken. First, short form scores were calculated for those participants who had completed the CESD-20 by summing only those ten items which occur on the CESD-10. For these same participants, the CESD-20 scores were regressed onto the CESD-10 scores to produce a regression coefficient estimating the association between the two forms (β = 1.77). Next, CESD-10 scores for those 35 participants who completed the shorter form were multiplied by that regression coefficient to produce estimated CESD-20 scores. The original CESD-20 scores for the 115 participants who completed the long form and the estimated CESD-20 scores for the 35 participants who completed the short form were used going forward (M = 8.84, SD = 9.60, Range: 0–46). The standard cutoff for clinically significant depressive symptoms on the CESD-20 is a score of 16 or above [71,72]. Based on these derived scores, 19% (n = 28) of the sample scored at or above the cutoff for clinically significant depressive symptoms. Finally, due to a large number of participants who endorsed no current depressive symptoms (n = 29), scores were non-normally distributed (skew = 1.40). Thus, all scores were transformed by taking log(*x*+1), resulting in more normal distribution (skew = -0.23) while maintaining the zero scores (Transformed Mean = 1.75, SD = 1.13, Range = 0–3.85).

### Statistical analyses

Prior to addressing our primary aims, we were interested in comparing parents’ reported dispositional and own-child specific empathy. To do so, we first calculated Pearson correlations across all subscales from the two measures. We also conducted paired samples *t*-tests to determine whether there were mean differences in parents’ reported dispositional versus child-specific empathy. To address our first and second aims, to examine the associations between depressive symptoms and dispositional and own-child specific empathy, respectively, Pearson correlations were calculated between depressive symptoms, dispositional empathy, and own-child empathy scores. Based on the equivocal findings concerning the associations between depression and the various empathy subscales, and the theory that decreased affective or cognitive empathy might be driven by heightened empathic distress, we further investigated whether the associations between depressive symptoms and the empathic concern and perspective taking subscales withstood when holding constant personal distress. To do so, we calculated partial correlations between both the affective and cognitive subscale scores from the dispositional and own-child empathy measures and depressive symptoms while controlling for personal distress. Our third and final aim was to examine potential differences between mothers and fathers in the associations between empathy and depressive symptoms. We first determined whether there were differences in either empathy or depressive symptoms across parent gender through independent samples *t*-tests. Next, we explored whether mothers and fathers showed the same pattern of results when comparing scores across the dispositional and own-child empathy measures by repeating the above described analyses separately within mothers and fathers. All analyses were completed using SPSS v. 23 (IBM Corp., Armonk, NY).

## Results

### Preliminary analyses

Pearson correlations between all subscales from the two empathy measures can be seen in Table 2. Scores on the empathic concern and perspective taking subscales were significantly positively correlated with each other both within and across the two scales. The associations with the personal distress subscale were more nuanced. Within the dispositional measure, personal distress was not related to either empathic concern or perspective taking, however within the own-child measure, personal distress was negatively related to empathic concern. Dispositional personal distress was positively correlated with own-child personal distress. Additionally, general personal distress was negatively related to own-child empathic concern. Results of the paired sample *t*-tests comparing dispositional and child-specific empathy showed that higher levels of empathic concern, *t*(149) = -7.78, *p* < .001, *d* = 0.64, 95% CI [-3.56, -2.11], perspective taking, *t*(149) = -2.11, *p* = .036, *d* = 0.17, 95% CI [-01.72, -0.06], and personal distress, *t*(149) = -3.79, *p* < .001, *d* = 0.31, 95% CI [-2.12, -0.67], were reported for one’s own child relative to dispositional.

### Associations between depressive symptoms and empathy

Pearson correlations between depressive symptoms, dispositional empathy, and own-child empathy scores can be seen in Table 3. Across both the dispositional and own-child empathy measures depressive symptoms were significantly negatively correlated with empathic concern and perspective taking and significantly positively correlated with personal distress. Thus, parents with greater depressive symptoms reported lower levels of empathic concern and perspective taking, but higher levels of personal distress both in their general tendencies and when considering their own child specifically. Approximately 20% of the sample met criteria for clinically significant depressive symptoms, allowing us to conduct exploratory analyses to examine whether a comparison of those below and above the CES-D cutoff differed in the manner expected based on the results produced from dimensional assessments of depressive symptoms. Results of a series of nonparametric Mann-Whitney *U*-tests revealed significant differences across both dispositional and own-child empathy in empathic concern (with those above the clinical cutoff scoring lower than those below: Dispositional *U*(122,28) = 1115.50, *p* = .004; Own-Child *U*(122,28) = 1220.00, *p* = .017) and personal distress (with those above the clinical cutoff scoring higher than those below): Dispositional *U*(122,28) = 764.00, *p* < .001; Own-Child *U*(122,28) = 956.00, *p* < .001). There was also a significant difference in *dispositional* perspective taking (with those above the clinical cutoff scoring lower than those below: *U*(122,28) = 1159.00, *p* = .007), but no difference for *own-child* perspective taking (*U*(122,28) = 1421.50, *p* = .167). Thus, aside from the lack of significant difference for own-child perspective taking, the results comparing those participants who met the criteria for clinical depression as compared to those who did not do indeed mirror the correlations between depressive symptoms and empathy.

**Table 3:** Pearson correlations between depressive symptoms and empathy scores.

	Dispositional			Own-Child		
	Empathic Concern	Perspective Taking	Personal Distress	Empathic Concern	Perspective Taking	Personal Distress
**Depressive symptoms**	-.37**	-.38**	.44***	-.37***	-.27**	.24**
	(-.36***)	(-.40***)		(-.33***)	(-.26**)	

*p < .05;

**p < .01;

***p < .001.

Partial correlations controlling for personal distress shown in parentheses. N = 150, df(partial) = 147.

Results of partial correlations between depressive symptoms and the empathic concern and perspective taking subscales when holding constant personal distress can be seen in Table 3. For both the dispositional and own-child empathy measures, depressive symptoms remained significantly correlated with both empathic concern and perspective taking scores even when holding constant personal distress.

Given the correlations between dispositional and own-child empathy scores were high, we sought to examine whether the association between depressive symptoms and own-child empathy could be explained by dispositional empathic tendencies or if depressive symptoms would be uniquely associated with own-child empathy. A series of linear regression analyses were conducted predicting parents’ empathy towards their own child from depressive symptoms (Table 4), while holding constant dispositional empathy on the corresponding subscale. Depressive symptoms remained a significant predictor of parents’ empathic concern toward their own child even after holding constant dispositional tendency for empathic concern; however, this association did not hold for perspective taking nor for personal distress.

**Table 4:** Regression models predicting own-child empathy from depressive symptoms, controlling for dispositional empathic tendency.

	Own-Child Empathy					
	Empathic Concern		Perspective Taking		Personal Distress	
Intercept	10.88*** (1.20)	13.03*** (1.54)	7.40*** (1.39)	8.33*** (1.85)	3.90*** (0.66)	4.43*** (0.73)
Dispositional Empathy	0.60*** (0.06) .65	0.55*** (0.06) .60	0.65*** (0.07) .59	0.62*** (0.08) .57	0.74*** (0.06) .72	0.79*** (0.06) .77
Depressive Symptoms		-0.66* (0.30) -.15		-0.29 (0.38) -.06		-0.56 (0.34) -.10
R^2^ Δ		.02*		.003		.01
R^2^	.43	.45	.35	.35	.52	.53
F stat	110.85***	59.30***	79.48***	39.93***	163.17***	83.90***

Regression coefficients are presented as: B (se) β. General empathy subscale scores used as a predictor in each model correspond with the predicted own-child empathy subscale. N = 150.

*p < .05

**p < .01

***p < .001.

### Comparing mothers and fathers

Our third and final aim was to examine potential differences between mothers and fathers in the associations between empathy and depressive symptoms. We first determined whether there were differences in either empathy or depressive symptoms across parent gender. Results of independent samples *t*-tests showed no difference in depressive symptoms across parent gender, *t*(148) = 0.40, *p* = .692, *d* = 0.07, 95% CI [-0.29, 0.44]. Mothers reported greater dispositional and own-child specific empathic concern and greater own-child specific perspective taking than fathers (Table 5).

**Table 5:** Comparison of dispositional and own-child empathy scores across parent gender.

	Mothers Mean (SD)	Fathers Mean (SD)	*t*	*p*	*d*	95% CI
**Dispositional Empathy**						
Empathic Concern	21.27 (5.45)	19.44 (5.57)	2.02	.045	0.33	0.04, 3.63
Perspective Taking	19.39 (5.51)	17.69 (5.23)	1.93	.055	0.32	-0.04, 3.44
Personal Distress	9.97 (5.70)	9.48 (6.23)	0.50	.618	0.08	-1.46, 2.44
**Own-Child Empathy**						
Empathic Concern	24.21 (4.82)	22.20 (5.23)	2.42	.017	0.40	0.37, 3.65
Perspective Taking	20.62 (5.47)	18.31 (6.08)	2.41	.017	0.40	0.42, 4.20
Personal Distress	10.77 (6.23)	11.33 (6.07)	-0.56	.580	0.09	-2.56, 1.44

df = 148.

There were a few differences across parent gender in regard to the correlations between subscales both within and across measures (Table 6). While in the whole sample analysis and within fathers there was no association between dispositional perspective taking and own-child personal distress, this association was moderately positive within mothers. Also, in the whole sample and within fathers there was a significant negative correlation between dispositional personal distress and own-child empathic concern, but this association was no longer significant within mothers. In the whole sample and within *mothers*, there was no association between dispositional empathic concern and dispositional personal distress, however this association was significantly negative within fathers. Among fathers, there was a moderately negative association between dispositional empathic concern and own-child personal distress, as well as between dispositional personal distress and own-child perspective taking, whereas these associations did not appear in the whole sample nor within mothers. All other associations were the same between mothers and fathers and did not vary from the whole group analyses. In other words, within mothers only greater dispositional personal distress was associated with lower own-child empathic concern and greater dispositional perspective taking was associated with greater own-child personal distress; whereas within fathers only greater dispositional empathic concern was associated with lower dispositional and own-child personal distress and greater dispositional personal distress was associated with lower own-child perspective taking.

**Table 6:** Correlations between subscales of dispositional and own child empathy within mothers and fathers, separately.

	**Dispositional**			**Own-Child**		
	Empathic Concern	Perspective Taking	Personal Distress	Empathic Concern	Perspective Taking	Personal Distress
**Dispositional**						
Empathic Concern	1	.77***	.02	.60***	.50***	.07
Perspective Taking	.63***	1	.04	.39**	.59***	.20
Personal Distress	-.22*	-.14	1	**-.13**	-.09	.70***
**Own-Child**						
Empathic Concern	.68***	.48***	-.38***	1	.52***	**-.15**
Perspective Taking	.52***	.57***	-.18	.58***	1	-.004
Personal Distress	**-.19**^†^	-.004	.75***	-.25*	-.07	1

^†^p < .10,

*p < .05;

**p < .01;

***p < .001.

Results for mothers are above the diagonal and results for fathers are below the diagonal. Results that differ in significance or direction between mothers and fathers or from the whole group analysis are highlighted in bold text. N(mothers) = 66; N(fathers) = 84.

We replicated the paired samples *t*-tests comparing scores across the two measures, for mothers and fathers separately. Both mothers, *t*(65) = -5.10, *p* < .001, *d* = 0.63, 95% CI [-4.09, -1.79], and fathers, *t*(83) = -5.86, *p* < .001, *d* = 0.64, 95% CI [-3.70, -1.82], reported significantly greater own-child specific empathic concern as compared to dispositional empathic concern. However mothers also reported greater perspective taking for their own child, *t*(65) = -2.01, *p* = .049, *d* = 0.25, 95% CI [-2.45, -0.005], and no differences on the personal distress subscales, *t*(65) = -1.39, *p* = .168, *d* = 0.17, 95% CI [-1.95, 0.35], whereas fathers did not differ on perspective taking, *t*(83) = -1.08, *p* = .286, *d* = 0.12, 95% CI [-1.75, 0.53], and did report lower dispositional personal distress as compared to own-child specific personal distress, *t*(83) = -3.94, *p* < .001, *d* = 0.43, 95% CI [-2.80, -0.92]. In other words, while both mothers and fathers reported higher levels of affective empathy for their own child as compared to dispositional affective empathy, only mothers reported higher levels of cognitive empathy and only fathers reported higher levels of empathic distress for their own child as compared to dispositional tendencies.

Finally, we examined whether the observed associations between depressive symptoms and either dispositional or own-child empathy differed or were consistent across mothers and fathers (Table 7). For fathers, the results mirrored those of the whole sample—depressive symptoms were significantly negatively correlated with the empathic concern and perspective taking subscales, and significantly positively correlated with personal distress across both dispositional and own-child measures. For mothers, these associations were similar for the dispositional measures of empathy, and for own-child perspective taking. However, the association between depressive symptoms and own-child empathic concern was only moderately negative, and there was no association with own-child personal distress. As was the case in the whole sample, the associations between depressive symptoms and either empathic concern or perspective taking, on either measure, did not change in direction or significance when holding constant personal distress.

**Table 7:** Pearson correlations between depressive symptoms and empathy scores within mothers and fathers, separately.

		Dispositional			Own-Child		
		Empathic Concern	Perspective Taking	Personal Distress	Empathic Concern	Perspective Taking	Personal Distress
**Depressive symptoms**	Mothers	-.29*	-.44***	.35**	-.23^†^	-.31*	.04
	Fathers	-.44***	-.36**	.50***	-.47***	-.27*	.38***

^†^p < .10;

*p < .05;

**p < .01;

***p < .001.

N(mothers) = 66; N(fathers) = 84.

Depressive symptoms remained a significant predictor of own-child empathic concern, over and above dispositional empathic concern, but only for fathers, B = −0.94, 95% CI [−1.70, -0.18], β = −.22, *t*(81) = −2.47, ΔR^2^ = .04, *p* = .016. Interestingly, and only for mothers, when holding constant dispositional personal distress, depressive symptoms significantly predicted own-child personal distress, B = −1.37, 95% CI [−2.47, -0.27], β = −.23, *t*(63) = −2.49, ΔR^2^ = .05, *p* = .015. Depressive symptoms did not predict own-child perspective taking over and above dispositional perspective taking, for either mothers or fathers. Analyses were conducted to examine interaction effects between gender and depressive symptoms. Despite the observed gender differences, the interaction term was not a significant predictor in the model predicting empathic concern, B = −0.49, 95% CI [−1.70, 0.72], β = −.12, *t*(125) = −0.80, ΔR^2^ = .003, *p* = .423. However, the interaction between gender and depressive symptoms was significant in the model predicting personal distress, B = 1.50, 95% CI [0.19, 2.82], β = −.28, *t*(125) = 2.27, ΔR^2^ = .02, *p* = .025.

## Discussion

The interactions a child has with his or her caregivers during the first few years of life are particularly impactful and have lasting effects on development across domains [1–3]. The current study was motivated by existing literature linking parental depression with significantly poorer outcomes in children; relations which are in part explained by differences in the parent–child relationship [4]. In the current study, we examine how parental depressive symptoms might impact their feelings of empathy toward their children as one potential explanation for how parental depression can impact the parent–child relationship. The aims in the current study were three-fold: (1) to examine the associations between depressive symptoms and dispositional empathy; (2) to explore the associations between depressive symptoms and empathy specifically toward one’s own child; and (3) to investigate how the association between depressive symptoms and empathy might be consistent or might differ across mothers and fathers. Within a sample of 150 parents of young children, we examined the associations among depressive symptoms, dispositional empathic tendencies, and empathy specifically toward their own children. We found evidence that (1) depressive symptoms are related to empathy, and the associations between depressive symptoms and the cognitive and affective types of empathy seem to exist above and beyond the association between depressive symptoms and personal distress; (2) depressive symptoms are related to lower levels of empathy towards one’s own child, beyond the effect of dispositional empathy, specifically in terms of empathic concern; and (3) the associations between depressive symptoms and empathy, both dispositional and toward one’s own child, vary depending on the parents’ gender.

Previous research on the link between depression and empathy have produced equivocal findings, specifically in terms of the association between depression and the three primary dimensions of empathy. Some studies have found that depression was related to lower cognitive and affective empathy but no association with empathic distress [e.g., 44], whereas others have found the opposite pattern such that depression was positively related to empathic distress but finding no association with cognitive or affective empathy [e.g., 46]. In line with the latter set of findings, it has been suggested that depression is in fact characterized by heightened empathy to such an extent that responding to others’ emotions results in potentially debilitating personal distress. As such, any deficits in empathy may be the consequence of a learned self-preservation technique [47,48]. If this were the case, we would expect that any association between depression and cognitive or affective empathy could be explained by differences in empathic distress. However, in the current study we found evidence more in line with the former set of studies [44,45], suggesting that depression would be negatively related to cognitive and affective forms of empathy and positively related to empathic distress. In this sample, depressive symptoms were indeed negatively related to both cognitive and affective empathy, and positively related to empathic distress. Critically, the associations with cognitive and affective empathy held even when accounting for empathic distress—suggesting that the association between depression and empathy is not simply an artifact of down-regulating empathic responses as a function of increased distress in reaction to others’ emotions. Rather, these findings indicate that depressive symptoms are uniquely associated with decreased empathic tendencies beyond any potential self-preservation processes, and are in line with the theoretical viewpoint that the relation between depressive symptoms and empathy is reflective of a more egocentric world-view [35,36].

However, it should be noted that the measurement of depression varies across the studies referenced here as well as in our own, specifically in terms of diagnosis versus symptoms. This is an important distinction, and it is possible that these associations operate differently for individuals with subclinical depressive symptoms as compared to those with a true diagnosis. Our exploratory analyses suggest that the results comparing individuals who did and did not meet the criteria for clinically significant depressive symptoms based on their CES-D scores mirrored those of the correlational analyses using depressive symptoms as a continuous measure. However, only a small portion of our sample (<20%) actually met the criteria; thus, these results should be interpreted with caution. Further research is needed to tease apart whether the inconsistent findings on the nuanced associations between depression and dispositional empathy might be in part driven by measurement differences.

The current study built on the existing work on the association between depression and empathy [e.g., 44–46] by exploring whether depression is also, and uniquely, related to empathy towards one’s own child. Indeed, the associations between depression and dispositional empathy replicated when looking at own-child specific empathy. Depressive symptoms were negatively related to parents’ reported cognitive and affective empathy toward their own child, and positively related to their reported empathic distress in regard to their own child’s emotions. Importantly, when accounting for dispositional empathy, this association remained significant for affective empathy (i.e., empathic concern). That is, for parents of young children, depressive symptoms are not only related to reduced empathy generally, but are associated with reduced empathy toward their own children over and above the association with dispositional empathy. This suggests that the observed links between parental depression and parenting behavior, and in turn child outcomes, may be mediated by differences in parents’ empathy toward their children.

The current study adds to the body of literature showing a link between parental empathy and child outcomes, explained in part by differences in parenting behavior [51,52]. Parents with depression might be at a heightened risk for exhibiting more negative parenting behaviors due, in part, to differences in empathy. Empathy is seen as an important contributor to mothers’ sensitive responses to their infants’ distress [73]. Mothers with higher levels of cognitive empathy are more likely to encourage perspective taking in their own children, and this behavior is in turn associated with higher levels of cognitive empathy in their children [51]. In a study using retrospective self-report, it was found that individuals who perceived greater empathy from their parents while growing up scored higher on measures of adaptive social-functioning [52]. While not a direct measure of empathy, maternal depression symptomology and diagnosis were both associated with blunted neural responses when observing their own infant’s emotional expressions [74]. Further, Humphreys and colleagues [75] found that maternal depressive symptoms were associated with increased self-focus (as measured by the types of pronouns used) in narratives about their child and their relationship. Further, this self-focus mediated the association between depressive symptoms and reduced warmth during an interaction with their child. Importantly, parents’ own-child specific empathy has been shown to predict increased risk of physical abuse and negative attribution of child behaviors [76]. Depression may also impact parents’ capacity to engage in dialogue about emotionally laden events with their children [32]. The current findings add to this body of literature and suggest that parental empathy may be a key target for parenting interventions. Parenting behavior in parents with depression can be positively impacted by appropriate intervention [77,78]. Emphasizing empathy for one’s own child may be particularly impactful for parents at risk for more negative parenting behaviors such as those with depression or postpartum depression, and might also be beneficial if incorporated into broader programs of treatment.

Our findings further emphasize the importance of considering these associations in conjunction with parent gender. That is, some of the overall effects we observed seem to have been driven by stronger associations either within mothers or fathers. We were unsure what to expect when considering parent gender in terms of the association between depression and empathy. However, previous work has shown that despite the imbalance in the number of studies examining the effects of maternal versus paternal depression, having a father with depression also has significant negative impacts on a child’s development [59,60]. In fact, studies on the epigenetic mechanisms linking parental psychopathology and child outcomes suggest that mothers and fathers may play a unique role [79]. In our sample, both mothers and fathers reported greater own-child specific affective empathy as compared to dispositional. However, mothers reported greater cognitive empathy for their own child whereas fathers reported no difference, and fathers report greater empathic distress for their own child as compared to dispositional whereas mothers reported no difference. That is, mothers reported thinking more about how their *child’s* emotions influence their behavior, as compared to their general tendency to do so, whereas fathers in our sample reported experiencing greater personal distress in response to their *child’s* emotions, again as compared to their general tendency to experience distress to others’ emotions. Additionally, we found that the way in which depression is associated with a parents’ empathy toward their own child differs for mothers and fathers. For fathers, the unique association between depressive symptoms and child-specific empathy, that is, above and beyond the effect of dispositional empathy, was found only for empathic concern—whereas for mothers this was the case only for personal distress. Together these findings suggest that how parents respond to their children’s emotional experiences and differentiate those responses from their dispositional empathic tendencies seems to vary across mothers and fathers, and further that the way depression can impact this process may also vary for mothers and fathers.

These findings are important to consider in light of research showing that mothers and fathers differ in the way they interact with their children. On average, fathers tend to engage in more negative parenting practices than mothers [80], while mothers tend to exhibit more sensitive parenting [81] and to talk about mental states and emotions with their children more than fathers [82]. Some of these behavioral differences may be driven by differences in the way mothers and fathers empathize with their children, which itself may be impacted by a parents’ experience of depression. Indeed, our findings suggest that fathers experiencing depressive symptoms may be less able, or less likely, to affectively empathize with their child in moments of heightened emotionality, while mothers experiencing depressive symptoms, on the other hand, may respond in the opposite way—internalizing their child’s emotion to the point that they experience their own distress. As such, their response in terms of parenting behaviors may also differ. Thus, research on the roles of depression and empathy in association with parenting behaviors and child outcomes would benefit from also considering how parent gender might moderate these effects. More research is needed to determine if the findings of this study replicate, as this is one of the first to examine these associations across parent gender.

Some limitations of the current study should also be noted. First, all of the constructs were assessed cross-sectionally and via parents’ self-report. Thus, it is important to interpret the associations between depressive symptoms and empathy in light of this. One implication of both the independent and dependent variables being measured through self-report is that the associations between empathy and depression could be influenced by shared method variance. It is also possible that some other, unmeasured underlying factor might contribute to an individual reporting both greater depressive symptoms and differences in empathic tendencies. Cross-sectional data preclude assumptions about the direction of the association between depressive symptoms and empathy. It is possible that individual differences in empathic tendencies put an individual at increased risk for developing depressive symptoms [83]. Further, we do not have a measure of parenting behavior—neither through self-report nor observation. Thus, our interpretation that empathy might be a path through which depression influences parent–child interactions and the early caregiving environment is not directly explored in the current study but is based on patterns of findings in the extant literature. We also did not explicitly ask or require that pairs of participants *not* be parents to the same children, thus it is possible that there is a limited nested nature to some of the responses that we have not accounted for. While we cannot be certain, we do have some level of confidence that this is not the case in our sample based on our method of removing potential “bots” and “farmers”. As noted in the Participants section, we removed responses with exact matches and repeats for IP address or geolocation (latitude and longitude). Thus, it is unlikely that participants were from the same household. However, this does not guarantee that no participants were parents to the same child. In addition, we did not ask participants to report the exact age of their child—only to confirm that they had a child in the target age range of 12 to 36 months. This is important to note in light of findings that the quality of the parent–child relationship and parenting behavior can vary greatly over time and at different developmental stages, especially in terms of negative parenting behaviors over the first years of life [84,85]. Further research is thus needed to examine the role of child age in the observed association between parental depressive symptoms and empathy toward their child. Lastly, as we have already mentioned, our measure of depression captured depressive symptoms and not diagnosis. Taxonomic analysis suggests that depression is more appropriately characterized in terms of a continuum of symptoms [86]. We did conduct an exploratory comparison of empathy scores across individuals who did and did not fall above the cutoff for clinically significant depressive symptoms, and these results largely mirrored those which used the continuous measure of depressive symptoms. However, only a small portion of our sample fell above the clinical cutoff, and these results may differ from those that used formal diagnostic criteria to group individuals.

In conclusion, we have extended the research on the links between depression and empathy, providing evidence that depression is related to all dimensions of empathy, that depression is associated with lower levels of empathy towards one’s own children, and that the associations between empathy and depression differ based on a parents’ gender. The current findings provide further indication that caregivers with elevated depression may engage in patterns of thoughts and behaviors that have implications for their interactions with their children. An individual’s empathy is shaped in part by their early caregiving experiences [87,88]. Experiencing parental depression is associated with poorer outcomes for children [13,14], particularly early in life [89]. Parents’ experienced empathy toward their own child may be one mechanism by which depression impacts the early caregiving environment. Thus, parent empathy towards their child may be an important target for clinical intervention in improving the early caregiving experiences for children at elevated risk for negative outcomes due to parental depression.
